# Anti-Proliferative, Analgesic and Anti-Inflammatory Properties of *Syzygium mundagam* Bark Methanol Extract

**DOI:** 10.3390/molecules25122900

**Published:** 2020-06-24

**Authors:** Rahul Chandran, Blassan P. George, Heidi Abrahamse

**Affiliations:** Laser Research Centre, Faculty of Health Sciences, University of Johannesburg, P.O. Box 17011, Doornfontein 2028, Johannesburg, South Africa; rahulc@uj.ac.za (R.C.); blassang@uj.ac.za (B.P.G.)

**Keywords:** lactate dehydrogenase, Hoechst stain, granuloma tissue, pain, inflammatory mediators

## Abstract

Cancer, pain and inflammation have long been a cause for concern amongst patients, clinicians and research scientists. There is an alarming increase in the demand for medicines suppressing these disease conditions. The present study investigates the role of *Syzygium mundagam* bark methanol (SMBM) extract against MCF-7 breast cancer cells, pain and inflammation. The MCF-7 cells treated with SMBM were analyzed for adenosine triphosphate (ATP), lactate dehydrogenase (LDH) levels, changes in cell morphology and nuclear damage. Hot plate, acetic acid and formalin-induced pain models were followed to determine the analgesic activity. Anti-inflammatory activity was studied using carrageenan, egg albumin and cotton pellet induced rat models. Microscopic images of cells in SMBM treated groups showed prominent cell shrinkage and nuclear damage. Hoechst stain results supported the cell death morphology. The decline in ATP (47.96%) and increased LDH (40.96%) content indicated SMBM induced toxicity in MCF-7 cells. In the in vivo study, a higher dose (200 mg/kg) of the extract was found to be effective in reducing pain and inflammation. The results are promising and the action of the extract on MCF-7 cells, pain and inflammation models indicate the potential of drugs of natural origin to improve current therapies.

## 1. Introduction

Cancer patients experience pain and inflammation during various stages of their disease [1]. As cancer progresses, patients experience more severe symptoms associated with inflammatory pain. The pain will depend on the type, stage and location of cancer. Hence, the management of pain and inflammation has been identified as one of the major tasks in cancer treatment. Various therapies targeting these risk factors have shown to counteract the cancer spread [2]. Non-steroidal anti-inflammatory drugs (NSAIDs) are among the widely used class of medicine against inflammation throughout the world. Long term use of NSAIDs drugs has been shown to reduce the incidence of cancer [3]. These drugs also suppress the microenvironment associated with inflammation in tumors and counteract the chemotherapeutic cancer pain [4]. Currently, these drugs have limited use, because of their adverse effects on bronchus, kidney, cardiovascular system, gastrointestinal lesion [5,6]. The interest in drugs with quick relief from pain and inflammation has become a major concern. Rodents are the most chosen animals for analyzing disease models, due to their genetic, anatomical and physiological similarities to humans. They are also preferred due to their reliability, being easy to handle, their rich behavioral profile, research efficacy etc. [7]. Hence, the investigations using medicinal plants in such research models could be much more effective against cancer, pain and inflammations. There is an increased interest in focusing the study of medicines of natural origin, especially plants because of their accessibility, minimum side effects and affordability [8].

*Syzygium mundagam* is a medicinal tree of the Myrtaceae family, endemic to Western Ghats. The plants of this family are being used for the treatment of several medical ailments including, asthma, diabetes mellitus, inflammation syndromes and bronchitis [9]. The plant is used in formulations with Tulsi against diabetes [10] and the fruits are eaten raw by the Kuruma and Paniya tribes of Wayanad [11]. To our knowledge, there are no reports on this plant being used for the treatment of cancer and related complications. Hence, an attempt was made to evaluate the antiproliferative, analgesic and anti-inflammatory properties of this plant. The relevance of this study will promote the traditional use and the significance of the genus *Syzygium* among the existing therapeutic medicinal plants against cancer, pain and inflammation.

## 2. Results

### 2.1. Cytotoxicity Analyses

#### 2.1.1. Cellular Morphology

**Figure 1:**
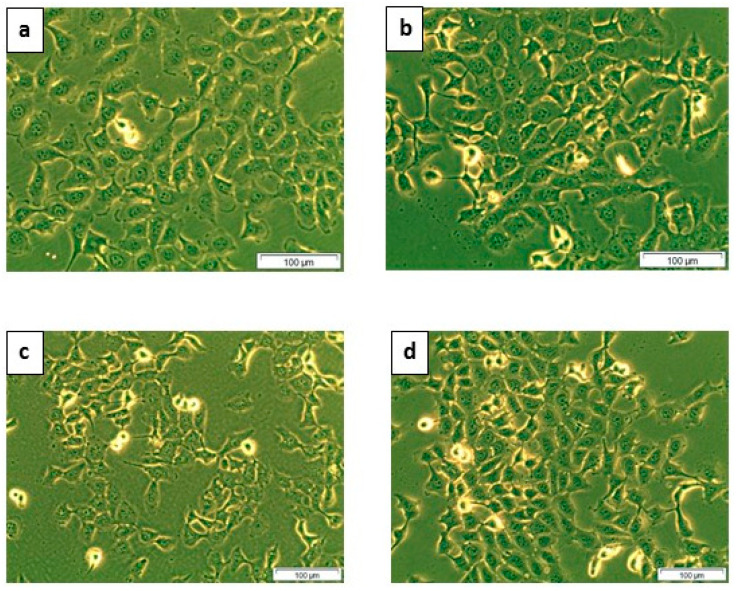
Morphology of MCF-7 cells after *Syzygium mundagam* bark methanol (SMBM) treatment. The cells in the figure signify the results of toxicity and death induced by SMBM (200× magnification). The cells in the control group (**a**) were seen healthy with an intact membrane and no signs of cell death. The difference in the number of dead rounded cells can be seen in groups treated with 10 (**c**) and 20 μg (**d**), compared to the control. Sub figure (**b**) represents 5 μg SMBM extract treated group.

The effect of *Syzygium mundagam* bark methanol (SMBM) doses on MCF-7 cells was evident after 24 h of incubation. The cells showed vacuolation, loss of membrane integrity, rounding, loss of cell to cell contact and detachment from the substratum. This indicates e cells SMBM induced toxicity in treatment groups (5, 10 and 20 μg) compared to the control, signifying the anti-proliferative property. The 5 μg dose was less effective compared to other doses (Figure 1).

#### 2.1.2. Adenosine Triphosphate (ATP) Cell Metabolism Assay

**Figure 2:**
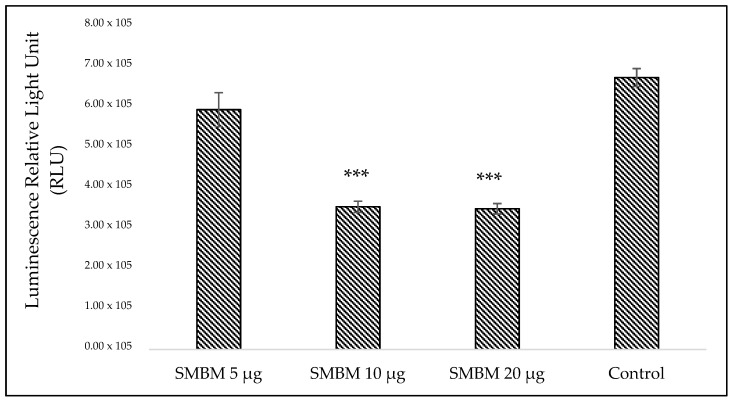
Adenosine triphosphate (ATP) metabolism in MCF-7 cells treated with SMBM. The higher ATP levels in the control group signify healthy and proliferating cells. However, the groups treated with different doses of SMBM extract show decreased ATP levels, which propose the antiproliferative nature of SMBM to MCF-7 cells. The doses at 10 and 20 μg showed almost similar toxic effects on MCF-7 cells. The data represent the mean ± SEM. Significantly different at *** *p* < 0.001 when compared to the control. SMBM—*S. mundagam* bark methanol extract.

The low levels of cellular adenosine triphosphate (ATP) directly implicate the decrease in the metabolic activities of cells. The extract caused a decline in the catabolic processes of MCF-7 cells, thereby reducing ATP production (Figure 2). The result also downgraded the rate of cellular proliferation, as depicted from the ATP levels of the groups treated with 10 (47. 53%) and 20 μg (47. 96%) of extract compared to the cells in the control group (Supplementary Figure S1; Percentage decrease in ATP proliferation treated with SMBM extract). A significant (*p* < 0.05) dose dependent cellular response was observed.

#### 2.1.3. Lactate Dehydrogenase (LDH) Assay for Cytotoxicity

**Figure 3:**
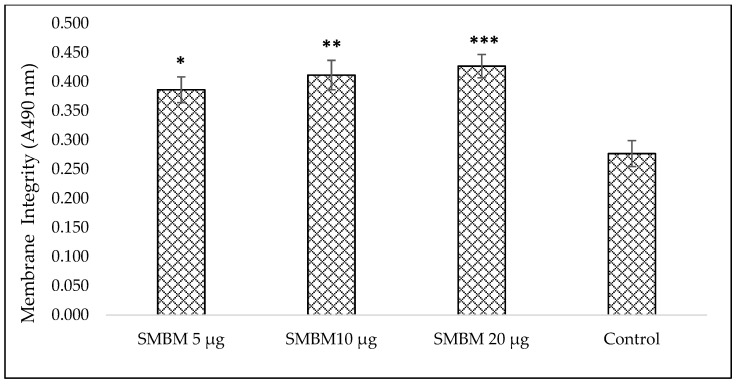
The amount of lactate dehydrogenase (LDH) released in MCF-7 cells treated with SMBM. The SMBM treated cells elevated the LDH release compared to the control. Among the groups, the maximum LDH release is seen in 20 μg SMBM treated cells. The data represent the mean ± SEM. Significantly different at * *p* < 0.05, ** *p* < 0.01 and *** *p* < 0.001 when compared to the control. SMBM—*S. mundagam* bark methanol extract.

The loss of membrane integrity and lactate dehydrogenase (LDH) leakage to the culture media were observed with slight variations among the extract treated groups. The membrane damage and LDH release from MCF-7 cells were measured using the CytoTox96^®^ Assay. The cellular damage caused by SMBM extract increased LDH release with increasing dose level (Figure 3). The cells treated with 20 μg extract induced maximum damage and increased the LDH release by 40.96%, compared to the control, whereas, 10 μg showed 34.05% increase in LDH (Supplementary Figure S2; percentage increase in LDH content treated with SMBM extract). The cell viability was calculated and IC_50_ of the extract was identified as 17 μg (Supplementary Figure S3; MCF-7 cellular viability treated with SMBM extract).

#### 2.1.4. Hoechst Stain

**Figure 4:**
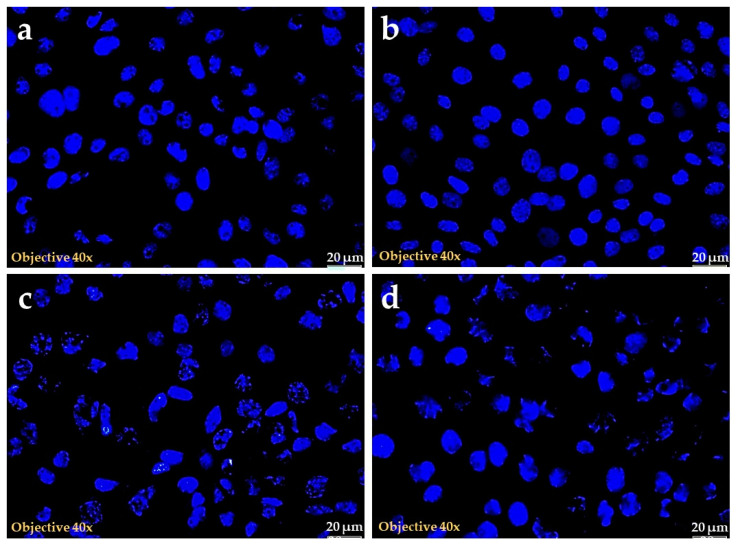
Hoechst nuclear stained MCF-7 cells after SMBM treatment. The groups represent (**a**) control, (**b**) SMBM 5 μg, (**c**) SMBM 10 μg and (**d**) SMBM 20 μg. The blue fluorescence in the figure confirms the nuclear binding of Hoechst stain. The cells treated with 5, 10 and 20 μg of SMBM shows evidence of dose dependant cell death compared to the control.

The nuclear stain using Hoechst implies the extend of nuclear damage caused by SMBM. The cells treated with extracts showed different levels of damage in a dose dependent manner. The nucleus stained uniformly signifying its dense nature in the control group. However, the 10 μg and 20 μg SMBM treated cells lost their nuclear integrity with irregular shape and nuclear condensation signifying the cell death, as shown in Figure 4. The lowest dose (5 μg) did not show morphological signs of toxicity in the test, with results similar to the control group.

### 2.2. Acute Toxicity Study

Animals fed with extract were monitored for the signs of toxicity through behavioral and morphological observations. The different dose levels of the extract proved to be safe even up to 2000 mg/kg. The extract didn’t alter the behavioral and morphological stature of the animals. Hence, 1/10th and 1/20th of the maximum dose was selected for analgesic and anti-inflammatory parameters.

### 2.3. Analgesic Test

#### 2.3.1. Acetic Acid Induced Writhing Test

In the acetic acid test, the writhing response was reduced significantly after SMBM treatment (Table 1). The analgesic activity observed with 200 mg/kg SMBM extract was close to aspirin (150 mg/kg) and inhibited the writhes to 48.76%. However, 100 mg/kg did not prove to be effective in pain reduction compared to the control.

#### 2.3.2. Formalin Induced Paw Licking in Mice

**Figure 5:**
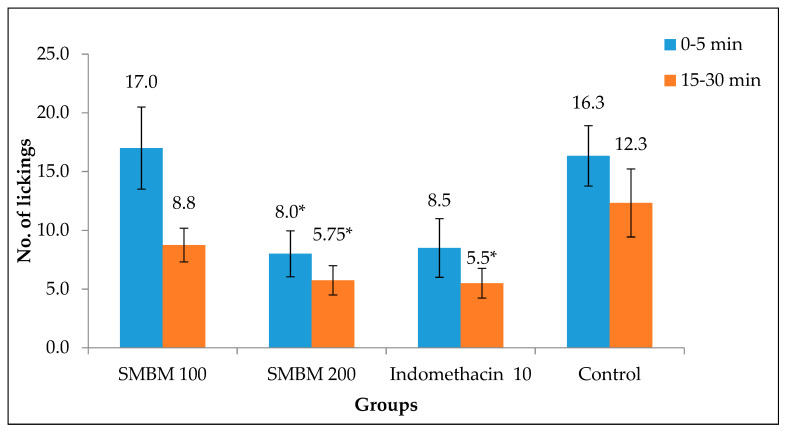
Paw licking response in mice treated with SMBM extract. The number of paw licking corresponds to the rate of pain response in mice. The results were significant at 200 mg/kg compared to the control. The data represent the mean ± SEM (n = 6). Significantly different at * *p* < 0.05 when compared to the control (untreated).

The effect of indomethacin and extract on the pain sensed by the central nervous system was accessed in this model. The results clearly depicted the ability of 200 mg/kg methanol extract to inhibit the pain sense effectively in the first (0–5 min) and second (15–30 min) phases of pain (Figure 5). The number of lickings was reduced to 5.75 in the second phase, where it was 8 in the first phase. This was very low compared to the control in both the phases.

#### 2.3.3. Hot Plate Method

The results in Table 2 show that the methanol extract increased the latency period at 200 mg/kg (10.50 ± 0.65 s). This shows the ability of extract to increase heat tolerance. There was a 40% reduction in heat sensitivity in mice (8.50 ± 0.87 s) with pentazocine (5 mg/kg) treatment. The results tabulated were obtained by comparing the final and initial latency period values.

### 2.4. Anti-Inflammatory Activity

#### 2.4.1. Carrageenan Induced Paw Oedema

The SMBM extract expressed a promising effect in this model. The maximum reduction in paw thickness was observed in the fourth hour, which was compared to the second hour measurement and tabulated (Table 3). The higher dose of SMBM (200 mg/kg) was much more effective (96.88%) than the 100 mg/kg dose and standard indomethacin (10 mg/kg). The significant inhibition differences can be observed among the groups at *p* < 0.05.

#### 2.4.2. Egg Albumin Induced Paw Oedema

The ability of SMBM extract to normalize the rat paw oedema was best observed with 200 mg/kg (77.97%). There was no much difference in the activity of extracts and indomethacin. In this model, 100 mg/kg SMBM extract also helped in reducing the paw inflammation by 68.31%. The reference drug indomethacin (10 mg/kg) was also effective (Table 4). The statistical significance was evident in 3rd h for 200 mg/kg, while it was in the fourth h for indomethacin.

#### 2.4.3. The Cotton Pellet Implanted Granuloma

On the eighth day of the study, a granulomatous accumulation was observed in cotton pellets removed from the axillary region of the rats. SMBM at 200 mg/kg prohibited the granuloma formation by 77.64%. This significantly (*p* < 0.001) reduced the weight of the cotton pellets implanted in extract treated groups compared to the control (Table 5). This result is a reflection of its activity against chronic inflammation.

## 3. Discussion

In this study, the authors have attempted to break the ‘cross-talk’ between pain, inflammation and cancer through in vitro and in vivo models. As discussed, targeting inflammatory mediators would check the progression of cancer and pain associated with it.

Studies have confirmed that natural products have the efficiency to alter protein functions involved in cancer progression [12]. The extract disrupted the membrane integrity and released LDH to the culture media. The significant increase in LDH levels with increasing doses of extract is in agreement with the reduced ATP levels, which clearly indicate the cytotoxic role of SMBM. The role of LDH in ATP production through the steps in pyruvate to lactate metabolism can be correlated to these levels in cell death determination. The ATP and LDH assay results reflect the antiproliferative effect of SMBM. The Hoechst stain helped in determining the DNA damage caused by the extract. The loss of shape, condensed nucleus, scattered nuclear granules and fragmented nucleus might have contributed to the morphological result observed in the study. Hence, the evident nuclear damage induced by the extract could be due to apoptosis, which has been proved earlier by several studies [13]. However, to support this, further studies are warranted. Syzygium spp. have been previously reported for their cytotoxic activity against various cancer cells. The essential oil extracted from *S. aromaticum* has shown interesting activity by inducing cell death in PC-3 and Hep G2 cell lines [14,15,16]. Eugenol and dehydrodieugenol could be among the active compounds that induce cancer cell death [17]. Similar activities were studied by Kouidhi et al. [18] and Kumar et al. [19] against breast, colorectal, leukemia and lung cancer cells. Another study performed using triterpenes isolated from *S. kusukusense* on breast (MCF-7), prostate (PC-3) and oral squamous (SCC2095) cell cancers reported their high potent cytotoxic activity with best depicted by betulinic acid (IC_50_, 5.7–7.6 μM) and ursolic acid (IC_50_, 1.7–3.7 μM) [20]. The methanol extract of *S. cumini* fruit skin inhibited proliferation and triggered cytotoxicity in HeLa and SiHac cells. The freeze dried fruit pulp also induced cell death in different breast cancer cell lines (MCF-7, MCF-10A, and MDA-MB-231) [21]. Ren et al. [22] studied the NF-κB and mitochondrial transmembrane potential inhibitory effects of ursolic acid (31 nM) isolated from *S. corticosum*. All these study reports reveal the cytotoxic properties of phenolic compounds. *S. Mundagam* has been previously quantified for the presence of total phenolics, tannins, gallic acid and quercetin, which might be taken into account for its activity against MCF-7 [23,24].

The central and peripheral analgesic activities were tested with the methanol extract in various animal models. The heat induced paw-licking is used for studying the stimulation-induced analgesia and mechanism of opioids [25]. For the central nervous system (CNS) pain sensitivity study, this model is considered as an ideal model to elevate the threshold levels of pain in mice towards heat [26]. In this method, the SMBM exhibited the ability to inhibit at higher doses and to suppress the pain sense through CNS on a moderate level. This study also proved effective against acetic acid and formalin induced peripheral analgesia. The intraperitoneal injection might have activated the visceral receptors of peritoneal activity sensitive to acetic acid [27], and results in the development of pain. The active compounds in the extract might have blocked this receptor activation to reduce pain.

The formalin test provides a clear platform to determine the moderate and continuous pain in animals through their characteristic early and late phases of pain [28]. The nociceptors, like Substance P, glutamate and bradykinin, which are produced in the first 5 min, are believed to be blocked by the active constituents of the extract of SMBM at a moderate level [29]. The activity of 200 mg/kg during the second phase may be because of the fact that the extract might have inhibited serotonin, histamine, bradykinin and prostaglandins, which are thought to be associated with inflammatory pain [30]. Hence, this study also supports the fact that the extract could also act effectively in inflammatory conditions. Chen et al. [31] in his study, reported that drugs targeting CNS acts equally on both phases, while Ahmadiani et al. [32] reported action of peripherally acting drugs only in the second phase. In the present study, even a higher dose of the extract could not show pronounced activity, compared to the positive controls like aspirin and indomethacin at low dose. The reason could be the purity of the standards and the mixture of compounds in the extract, which not only elevates the activity, but sometimes may reduce due to some antagonism among the compounds. The active phenolic compounds gallic acid and quercetin reported previously in this plant [23] might have resulted this activity. Similarly, several other compounds isolated from *S. cumini* leaves, myricetin, quercetin, acetylated flavonol glycosides, triterpenoids and tannin have also been reported to possess analgesic property [33,34]. The analgesic activity shown by *S. samarangen* can also be correlated to the present study [35]. Taher et al. [36] also reported analgesic activity of kaempferol isolated from *S. cumini* leaves. The crude hydroalcoholic extract of the leaves (100–300 mg/kg) also reduced the pain in hot plate and formalin induced analgesic models.

The oedema developed by carrageenan and egg albumin may be classified into three phases: with the release of histamine and serotonin, the early phase (the first 90 min) is initiated; bradykinin release marks up the second phase (90–150 min); and the third phase (after 180 min) is mediated by prostaglandin [37]. From the results, it is clear that the extract could possibly act by inhibiting the action of kinin or prostaglandins more significantly in the late stage rather in the early stages of inflammation, whereas the extract responded moderately in egg albumin induced model with less differences in the paw. The study model with egg albumin couldn’t bring up much inflammatory condition in rats compared to the carrageenan as studied in *S. cumini* [9] and *S. callophyllifolium* [38] bark extract, showed a highly promising ability to cure different inflammatory conditions.

Cotton pellet implanted inflammation model helped us to assess the action of the plant extract against chronic inflammatory conditions. Chronic inflammation can result from acute inflammation or without an acute phase. Essentially, formation of granulation tissue is a feature in chronic inflammatory phase. The granulation tissue comprises new blood vessels, fibroblasts and extracellular matrices. The injury and cotton implantation produces granuloma over course of time, which contains neutrophils, inflammatory cells and fibroblasts. It is evident from the study that the extract has the ability to block the formation of granulomatous tissue. As in the other models, the higher dose of the bark methanol extract was very effective in inhibiting granuloma formation with an inhibition of 77.64%. This indicates the capacity of SMBM extract to hinder the abnormal permeability of the blood capillaries and migration of inflammatory cells. The activity of SMBM extract could be due to phenolic and flavonoid compounds [23,24], reported by the authors earlier. Essential oil and oleanolic acid extracted from the flower buds of *S. aromaticum* have been well studied for pharmacological properties, including the ability to inhibit nociception and inflammation [39,40].

The ability of active ingredients, such as flavonoids and phenolics, to inhibit eicosanoid, such as prostaglandins, can be taken up as a promising factor of reducing inflammation and pain in cancer patients. Further research will help to study the role of pain and inflammatory mediators in the progression of cancer.

## 4. Materials and Methods

### 4.1. Collection of Plant Materials

The bark was collected during the month of October 2012 from Wayanad district of Kerala, Western Ghats. The collected plant material was identified and the authenticity of *S. mundagam* was confirmed by Dr. M. Prabhukumar, Scientist, Plant Systematic and Genetic Resources Division, Centre for Medicinal Plant Research, Arya Vaidya Sala, Kottakkal, Malappuram, Kerala and deposited (CMPR 7932). Freshly collected plant material was cleaned to remove adhering dust, and then dried under shade. The dried sample was powdered and used for further studies.

### 4.2. Successive Solvent Extraction

The air dried, powdered tree bark (100 g) was extracted in a Soxhlet extractor using methanol (300 mL). The extract was then dried in rotary vacuum evaporator and stored for further in vitro and in vivo studies. The extract thus obtained was dissolved in deionized water at a ratio of 1:1 (*w*/*v*) for in vitro studies.

### 4.3. Cytotoxicity Analyses

#### 4.3.1. Cell Culture and Treatment

A commercially purchased MCF-7 breast cancer cell line (ATCC HTB-22) was used for the cytotoxicity study. Approximately, 5 × 10^5^ cells were seeded in 3.4 cm diameter culture dishes and cultured in Dulbecco’s modified Eagle’s media (DMEM, Sigma-Aldrich, D 6429, Modderfontein, South Africa), supplemented with 10% foetal bovine serum (FBS, Gibco, 306.00301), 1% antifungal (amphotericin-B, Gibco, 104813) and 1% penicillin and streptomycin (Sigma Aldrich: P4333). The culture was maintained at 37 °C with 5% CO_2_ and 85% humidity for 4 h, to allow the cells to attach. Culture dishes with more than 90% confluence were used for experiments. The experimental groups were divided into untreated control and cells treated with SMBM at three different doses, 5, 10 and 20 μL (i.e., 5, 10 and 20 μg).

#### 4.3.2. Cellular Morphology-Inverted Microscopy

The morphology of the SMBM extract (5, 10 and 20 μg) treated cells was analysed after 24 h of incubation using an inverted light microscope (Wirsam, Olympus CKX41, Johannesburg, South Africa Once digital images were recorded, cells were trypsinized using 1 mL/25 cm^2^ of TrypLE Express (Gibco-12563-029 (ThermoFisher Scientific), Johannesburg, South Africa) and re-suspended in Hank’s Balanced Salt Solution (HBSS) to perform biochemical assays.

#### 4.3.3. Cellular Proliferation-Adenosine Triphosphate (ATP) Luminescent Assay

The CellTiter-Glo1 luminescent assay (Promega, G7571, Anatech Analytical Technology, Bellville, South Africa) is a homogeneous method for determination of cellular proliferation and quantification of ATP present in metabolically active cells. An equal volume (50 µL) of reconstituted ATP reagent and the cell suspension was mixed on a shaker for 2 min to induce cell lysis, followed by incubation at room temperature for 10 min in the dark to stabilize the luminescent signal. The luminescent signal was read using the 1420 multilabel counter victor3 (Perkin-Elmer, Separation Scientific, Johannesburg, South Africa).

#### 4.3.4. Cytotoxicity-Lactate Dehydrogenase (LDH) Assay

The membrane integrity was assessed by estimating the amount of LDH present in the culture media. The cytosolic enzyme LDH will be released into the media due to membrane damage. The Cyto-Tox96 X assay (Anatech, Promega G 400, Bellville, South Africa) was used to measure the LDH released. An equal volume (50 µL) of reconstituted LDH reagent and cell culture medium was mixed and incubated in the dark at room temperature for 30 min. The colorimetric compound was measured spectrophotometrically at 490 nm (Perkin–Elmer, VICTOR3™, Midrand, South Africa).

#### 4.3.5. Hoechst Stain

The damage caused by SMBM extract was analyzed using Hoechst nuclear stain. The MCF-7 cells were cultured in 3.4 cm^2^ diameter culture dishes over sterile cover slips and allowed to reach above 80% confluence The cells were then treated with different concentrations of SMBM (5, 10 and 20 μg). After 24 h incubation, cells were stained with 1 μg/mL Hoechst stain (Hoechst 33258, H21491, Invitrogen (ThermoFisher Scientific), Johannesburg, South Africa) for 30 min. Thereafter, the cells were rinsed with PBS and the blue fluorescent signal was examined using the Olympus fluorescent microscope (Johannesburg, South Africa).

### 4.4. Animals and Ethical Approval

Both Swiss albino mice and Wistar albino rats used in the present study were procured from the animal house unit at the Nandha college of Pharmacy, India. Healthy Wistar albino rats (100–150 g) and Swiss albino mice (20–25 g) of either sex and of approximately the same age, were used for the study. They were fed with standard chow diet and water ad libitum. The animals were housed and aclamatized in polypropylene cages in a well maintained and clean environment (28 °C) for 15 days prior to the study. The experimental protocol was subjected to scrutiny of the institutional animal ethics committee, according to Committee for the Purpose of Control and Supervision of Experiments on Animals (CPCSEA) for experimental clearance (No.688/02/C/CPCSEA). The animals were euthanized after the study period and were incenerated as per the institutional standard biosafety protocol.

### 4.5. Acute Toxicity

Acute oral toxicity studies were performed [41] according to the Organization for Economic Co-operation and Development (OECD) guidelines. Male Swiss albino mice (n = 6/each dose) were selected for acute toxicity study. The animals were fasted overnight with free access to water. *S. mundagam* bark methanol (SMBM) extract (suspended in 0.6% Carboxy Methyl Cellulose (CMC)) was administered orally at a dose of 5 mg/kg. The general behaviors such as motor activity, tremors, convulsions, straub reaction, aggressiveness, piloerection, loss of lighting reflex, sedation, muscle relaxation, hypnosis, analgesia, ptosis, lacrimation, diarrhoea and skin colour were observed for 3 days. If mortality was observed in 4/6 or 6/6 animals, then the dose administered was considered as toxic. Since no mortality or signs of toxicity were observed, the experiment was repeated with doses 100, 200, 500, 1000 and 2000 mg/kg. All of the test was carried out on caged animals, following standard protocols for route of administration.

### 4.6. Analgesic Test

#### 4.6.1. Acetic Acid Induced Writhing Test

The peripheral analgesic activity of SMBM was evaluated in male Swiss albino mice using the acetic acid-induced writhing test [42,43]. In the writhing test, mice (n = 6/group) in group I and II were orally administered with SMBM (100 and 200 mg/kg respectively) extract before 1 h of intraperitoneal injection of acetic acid (0.6%, 10 mL/kg). The control and standard group orally received (0.6%,1 mL) CMC and aspirin (150 mg/kg), respectively. The number of writhing reflex was counted for the next 15 min.

#### 4.6.2. Formalin-Induced Paw Licking in Mice

The test was carried out in Swiss albino male mice, according to the method described earlier [29]. Each group was divided into 6 mice each. 0.6% CMC (1 mL) was orally administered as a normal control and indomethacin (10 mg/kg, i.p.) was used as a standard drug. Twenty microliters of 1% formalin was injected into the dorsal surface of the left paw of mice 1 h after oral administration of the vehicle, standard and SMBM (100 and 200 mg/kg groups, p.o.). The duration of paw licking(s) was monitored between 0–5 min (first phase) and 20–25 min (second phase) after formalin challenge.

#### 4.6.3. Hot Plate Method

This method was used to evaluate the analgesic activity (delay in latency of pain response) according to the procedure described earlier in Swiss albino male mice [44]. The groups were divided into 6 mice each. The mice were treated orally with different doses of SMBM (100 and 200 mg/kg groups, p.o.). After 1 h, the animals were placed on a hot plate maintained at 55 ± 0.5 °C. The reaction time was recorded as per the time taken by the animals to blow or lick the fore or hind paw or jump out of the plate. A total of 0.6% CMC (1 mL) was used as an untreated control, and pentazocine (5 mg/kg, i.p.) was used as a reference drug.

### 4.7. Anti-Inflammatory Activity

#### 4.7.1. Carrageenan Induced Paw Oedema

The anti-inflammatory effect of methanolic extract of bark was assessed in the acute inflammation method already described [45]. The initial, normal paw thickness of each Wistar albino male rat was noted. Rats were orally administered with the different doses of the extract (100 and 200 mg/kg, p.o.). One hour later 0.1 mL of 1% carrageenan suspension was injected into the sub plantar region of the right hind paw of each rat. The control rats orally received a 0.6% CMC suspension (1 mL), while indomethacin (10 mg/kg) was used as standard drug. Increase in linear paw thickness was taken as a measure of oedema.

#### 4.7.2. Egg Albumin Induced Paw Oedema

Oedema was induced by subplantar injection of 0.1 mL of 1% (*w*/*v*) egg albumin [46] into the right hind paw of Wistar albino male rats. The paw thickness of the control, standard and plant extract treated rats were measured every hour up to 4 h after the administration of egg albumin. The oedema size was measured with a Vernier calliper. Different groups of animals were pre-treated with SMBM (100 and 200 mg/kg extract suspended in 0.6% CMC) and indomethacin (10 mg/kg). The drugs and standard were administered orally 1 h before eliciting paw oedema.

#### 4.7.3. The Cotton Pellet Implanted Granuloma

The animals were divided into four groups of six Wistar albino male rats each. After shaving the fur, the animals were anesthetized. Sterile, pre-weighed cotton pellets (50 ± 1 mg) were implanted in the axilla region of each rat [47]. The plant extracts (100 and 200 mg/kg) and standard indomethacin (10 mg/kg) were administered orally to the respective group of animals for seven consecutive days from the day of cotton pellet implantation. On the eighth day, the animals were anesthetized again; the cotton pellets were removed surgically and made free from extraneous tissues. The pellets were incubated at 37 °C for 24 h and dried at 60 °C. The weight of the granulomatous tissue was calculated by the difference between the initial and the final dry weight of the cotton pellets.

### 4.8. Statistical Analysis

All the results were expressed as mean ± SEM (n = 6). Statistical significance was determined by using the one way ANOVA, followed by Dunnett’s multiple comparison tests. *p* < 0.05 was considered statistically significant.

## 5. Conclusions

The results of this study represent an obvious role of *S. mundagam* in inducing toxicity in MCF7 breast cancer cells. Moreover the extract has also reduced pain and inflammation in animal models. The extract altered morphology, ATP and LDH contents of the cells supporting the inhibition of cell proliferation and cell death. An effective anti-inflammatory potential was also observed with active inhibition against central and peripheral pain. The active phenolic compounds in the extract could be responsible for these activities. The study results will encourage the use of *S. mundagam* bark methanol extract in formulating drugs with multiple healing properties.

## Figures and Tables

**Table 1:** SMBM activity in acetic acid-induced writhing test.

Groups	Doses (mg/kg b. wt.)	No. of Writhes (in 30 s)	% Inhibition
SMBM	100	28 ± 3.49	7.44
SMBM	200	15.5 ± 2.75	48.76
Control		30.25 ± 9.73	
Aspirin	150	14.5 ± 1.19	52.07

The data represent the mean ± SEM (n = 6). The results were not significant at * *p* < 0.05 when. The data represent the mean compared to normal (untreated), SMBM—*S. mundagam* bark methanol extract; b. wt.—body weight.

**Table 2:** Effect of SMBM against hot plate method induced pain.

Groups	Dose (mg/kg b. wt.)	Latency Period (in sec)						% Inhibition
		0	30	60	90	120	150	
Control	-	3.50 ± 0.30	6.00 ± 0.40	5.30 ± 0.60	8.80 ± 2.1	4.80 ± 1.3	6.50 ± 0.6	-
SMBM	100	2.25 ± 0.25	4.75 ± 0.85	4.00 ± 0.41	6.00 ± 0.71	4.75 ± 0.85	8.50 ± 0.29	52
SMBM	200	3.50 ± 0.29	4.25 ± 0.63	6.75 ± 0.48 *	4.25 ± 0.48	6.25 ± 1.55 *	10.50 ± 0.65 ***	57.14
Pentazocine	5	3.50 ± 0.29	3.75 ± 0.25	4.25 ± 0.25	4.50 ± 0.96	5.00 ± 0.41	8.50 ± 0.87	40

The data represent the mean ± SEM (n = 6). Significantly different at * *p* < 0.05, *** *p* < 0.001 when compared to normal (untreated) SMBM—*S. mundagam* bark methanol extract; b. wt.—body weight.

**Table 3:** Activity of SMBM against carrageenan induced oedema model.

		Paw Thickness (mm)					
Groups	Dose (mg/kg b. wt.)	0th h	1st h	2nd h	3rd h	4th h	% Inhibition
Control	-	3.97 ± 0.15	6.27 ± 0.27	6.12 ± 0.85	5.93 ± 0.35	6.09 ± 0.84	-
SMBM	100	4.28 ± 0.20	6.39 ± 0.18	7.02 ± 0.45	6.42 ± 0.40	6.32 ± 0.76	95.19
SMBM	200	4.27 ± 0.15	6.74 ± 0.16	7.11 ± 0.77	6.68 ± 0.77	6.04 ± 0.84 *	96.88
Indomethacin	10	4.29 ± 0.41	6.05 ± 0.15	7.12 ± 0.31	6.35 ± 0.32	6.10 ± 0.45 *	96.71

The data represent the mean ± SEM (n = 6). Significantly different at * *p* < 0.05 when compared to normal (untreated) SMBM—*S. mundagam* bark methanol extract; b. wt.—body weight.

**Table 4:** Effect of SMBM against egg albumin induces paw oedema in rats.

Groups	Dose (mg/kg b. wt.)	Paw Thickness (mm)					
		0 h	1 h	2 h	3 h	4 h	% Inhibition
Control		4.44 ± 0.06	5.63 ± 0.22	5.86 ± 0.26	5.97 ± 0.45	5.67 ± 0.16	
SMBM	200	4.61 ± 0.14	6.00 ± 0.61	5.93 ± 0.54	4.67 ± 0.43 *	5.05 ± 0.48	77.97
SMBM	100	4.62 ± 0.25	5.07 ± 0.14	5.36 ± 0.34	4.96 ± 0.15	4.75 ± 0.42	68.31
Indomethacin	10	5.03 ± 0.30	5.54 ± 0.28	4.88 ± 0.29	5.55 ± 0.43	4.38 ± 0.40 *	61.33

The data represent the mean ± SEM (n = 6). Significantly different at * *p* < 0.05, when compared to normal (untreated) SMBM—*S. mundagam* bark methanol extract; b. wt.—body weight.

**Table 5:** Activity of SMBM against cotton pellet induced granuloma model.

Groups	Dose (mg/kg b. wt.)	Initial Pellet wt (mg)	Mean wt on 8th Day (mg)	Mean Increase in Pellet wt (mg)	% Inhibition
Control	-	50	129.00	79 ± 3.61	-
SMBM	100	50	121.00	71 ± 5.20	10.13
SMBM	200	50	67.77	17.67 ± 2.34 ***	77.64
Indomethacin	10	20	83.33	33.33 ± 2.73 ***	57.81

The data represent the mean ± SEM (n = 6). Significantly different at *** *p* < 0.001 when compared to normal (untreated) SMBM—*S. mundagam* bark methanol extract; b. wt.—body weight.

## Supplementary Materials

The following are available online. Figure S1: Percentage decrease in ATP proliferation treated with SMBM extract; Figure S2: Percentage increase in LDH content treated with SMBM extract; Figure S3: MCF-7 cellular viability treated with SMBM extract.
