# Supplementary material for: Anti-Proliferative, Analgesic and Anti-Inflammatory Properties of Syzygium mundagam Bark Methanol Extract
Source: Molecules. 2020 Jun 24;25(12):2900. doi: 10.3390/molecules25122900 (PMC7355416; doi:10.3390/molecules25122900)
Supplement: Supplementary file 1 [file molecules-25-02900-s001.pdf]

Figure S1. Percentage decrease in ATP proliferation treated with SMBM extract

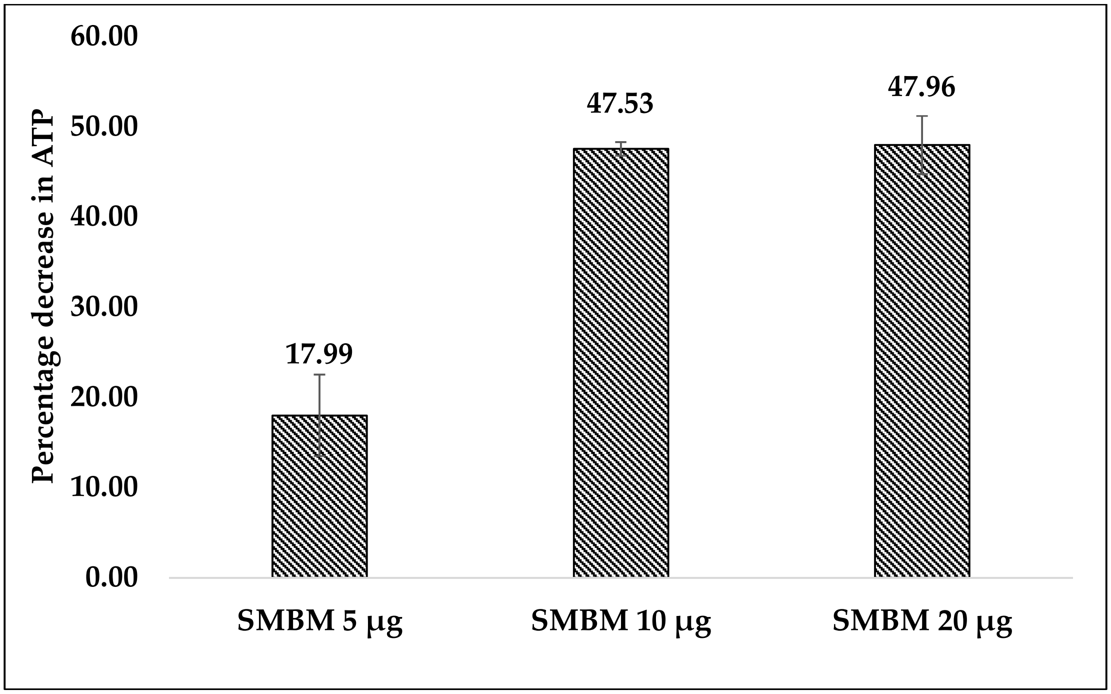

The data represent the mean ± SEM. SMBM- *S. mundagam* bark methanol extract

Figure S2. Percentage increase in LDH content treated with SMBM extract

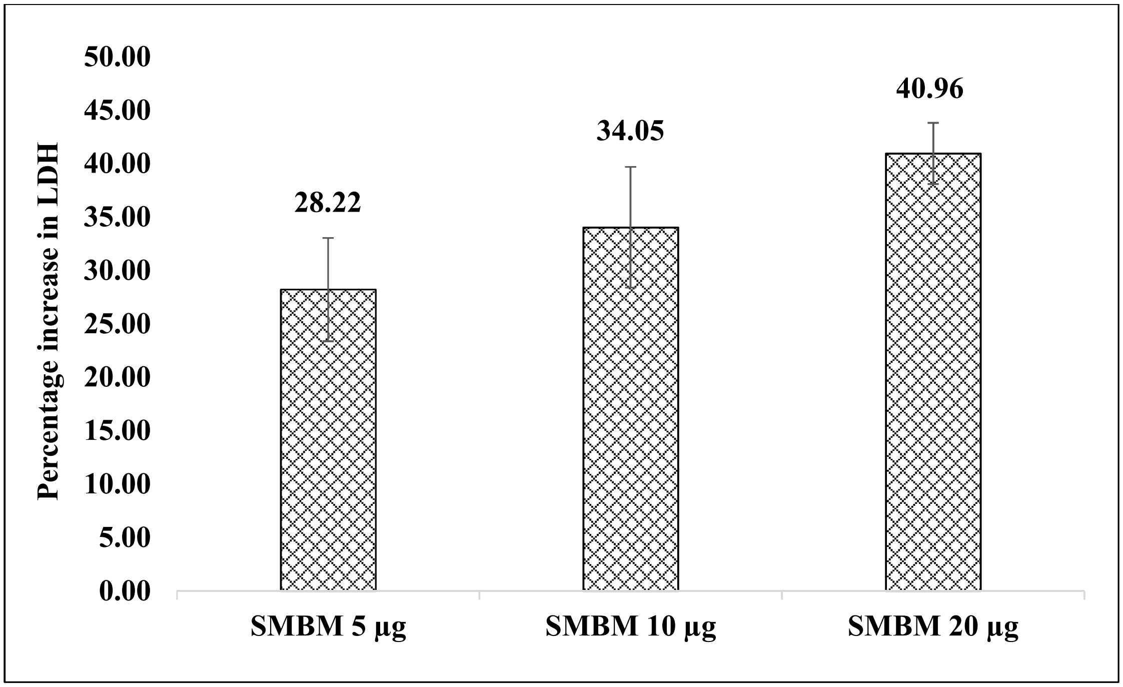

The data represent the mean ± SEM. SMBM- *S. mundagam* bark methanol extract

Figure S3. MCF-7 cellular viability treated with SMBM extract

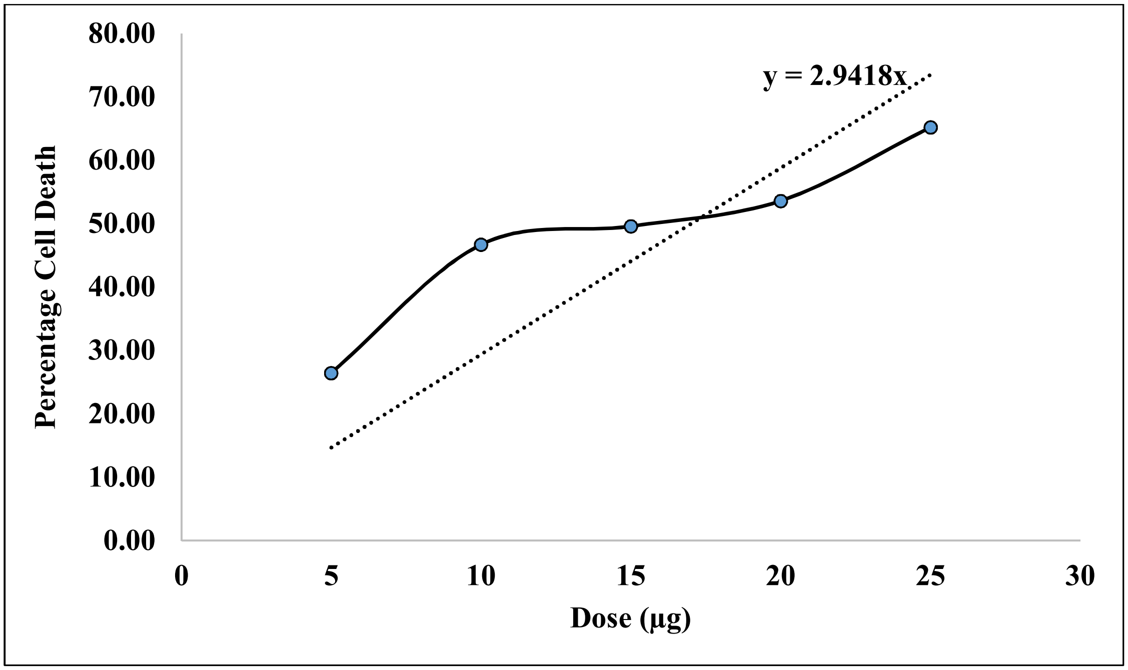

The figure represent dose dependent cell death induced by the SMBM extract. SMBM- ***S. mundagam*** **bark methanol extract**
